# On the Exhibition of Quinine in the Febrile Paroxysm

**Published:** 1851-03

**Authors:** Otis F. Manson

**Affiliations:** Granville co., N. C.


### SELECTIONS

FROM

### FOREIGN AND HOME MEDICAL JOURNALS.

---

*On the Exhibition of Quinine in the Febrile Paroxysm.* By Otis F. Manson, M.D., of Granville co., N. C.—The introduction of quinine as a remedy in the treatment of remittent fever, *given in the remission*, was perhaps coeval with its discovery as one of the constituents of cinchona, the bark itself having been used in that stage of the disease by Lind, Senac, Clark, Balfour and others, anterior to its disintegration; but it was perhaps not more than twenty-five years ago that this salt was administered in the exacerbation, and at this time the practice is almost exclusively confined, in our own country, to the extreme southern and western States.

A number of my professional friends in Virginia and North Carolina having adopted this plan of treatment, at my suggestion, I have felt it my duty, in compliance with their solicitations, to place within their reach all of the knowledge I possess on the subject, drawn from my own experience and many others; and I therefore embrace the opportunity to do so, offered by your flattering invitation to contribute to the pages of your journal.

Remitting fever has for many years assumed a congestive character, in the vast majority of instances being attended with the periodical recurrence of a stage of congestion, commonly termed chill; this stage being characterized by a diminution of temperature, varying from a slight coldness at the tips of the nose, fingers, and toes, to a fearful frigidity of the whole extremities, and in some few instances a death-like coldness of the whole surface of the body; these latter cases being termed, *par excellence*, "congestive fever."

Although in many instances this stage is not manifested after the initial chill, by any discernable reduction of temperature in any part of the body for several days, yet it sooner or later becomes apparent to the touch of the attendant, although unfelt by the patient, who, instead of experiencing any sensation of cold, is oppressed with intense heat and unquenchable thirst; indeed, we have often discovered the presence of this stage by the increased urgency of this thirst and desire for removal of the clothing.

At the same time that this coldness of the extreme parts is perceived, *the rest of the body is of high febrile heat;* the pulse quick, frequent and small, the breathing laborious, deep sighing, oppression at the præcordia, pain in the head, loins and limbs, anxiety, nausea and vomiting.

After a lapse of time, varying according to the violence of the attack, but averaging about an hour, this stage terminates in the exacerbation, the heat becomes now diffused over the whole surface, the pulse becomes diminished in frequency, and increased in fulness and force, the pains and thirst still continuing, occasionally, though not commonly attended with delirium and other indications of cerebral disturbance; pain on pressure of any of the abdominal regions not generally present in the first exacerbation, but more apparent as the disease advances, especially in the epigastric region. This stage, after a lapse of 24 or 48 hours, according to the type of the disease, is succeeded by a recurrence of the stage of congestion; the tongue now becomes sensibly altered, but so variable in appearance that no particular description will apply to it. Evidences of organic irritation in the great cavities, particularly of the stomach, bowels and liver, now become more apparent, and the abdomen becomes tense, tender, and in some cases meteoric. From the fifth to the fifteenth day, the disease terminates, without aid, in 1st, intermittent fever; 2d, fever of a more continued character; 3d, death.

I have deemed it necessary to give this brief description of this disease as it usually appears in my field of observation, in order that the following remarks may be fully understood, as the object of the article is chiefly to display the effects of quinine in this disease.

Given in free and full doses of from twenty to thirty grains in the exacerbation, quinine reduces the pulse in frequency, fulness and force; it removes local determination to the head, chest and abdomen, thereby relieving pain, restlessness, nausea, vomiting and diarrhœa, producing generally a copious, warm diaphoresis, and, in short, to expel fever and

——— "all its dire attendant train,"

safely, surely and expeditiously, in a period of time varying from four to ten hours from its administration.

But along with the employment of quinine the following means are resorted to:

Called to a patient in the exacerbation, venesection is practised in every case where the pulse will justify it, but it is rarely that this is called for. In fact, the general experience of myself and *confreres* is adverse to the use of the lancet in this affection; the pulse, though often full, and apparently tense, is generally *compressible.* Local bleeding by cups and leeches will usually be sufficient, and if there is tenderness on abdominal pressure, or other symptoms of visceral implication, these should be freely applied. Should symptoms of cerebral irritation be present, a copious flow of blood will be obtained by cups to the mastoidal regions.

This is to be immediately followed by a cathartic dose of calomel, say twenty grains, and in three hours thereafter by twenty grains of quinine, diffused in a wineglassfull of cold water, and if the febrile excitement is intense, thirty grains may be given, but twenty grains will in almost every instance be sufficient for the first dose. Four hours are now suffered to elapse, during which the local bleeding is continued, if required. In this time, in a vast majority of instances, the fever will be considerably reduced, when the quinine should be repeated in doses of six to ten grains every four hours, until thirty-five or forty grains are taken. If in four hours after the first dose has been taken the fever has not sensibly declined, the large dose (twenty grains) may be repeated; but in many hundred cases the first dose has seldom failed in that time to produce an evident declension of the fever, rendering the after employment of the medicine, in doses of six to ten grains, amply sufficient to effect a complete solution of the disease, the great aim being to throw into the system at least thirty-five or forty grains before the period for the recurrence of the next paroxysm.

In cases attended with obstinate vomiting the stomach should be previously calmed by the administration of forty or fifty drops of laudanum, and if this should be rejected the laudanum may be given by the rectum in double that quantity. But if the gastric irritability should not yield to the anodyne, after local depletion also has been employed as before advised, (and in some cases the stomach will not bear the presence of any medicine long enough to induce its effects,) the quinine must be given by the rectum. One drachm of quinine should be suspended in four ounces of mucilage of gum arabic or thin starch, and given at once as an enema; to be repeated at the same intervals as *per orum*, and in thrice the quantity above recommended.

If, however, the stomach will bear the quinine for an hour, the nausea and vomiting will in almost every instance cease.

In some few instances, although the foregoing treatment has been adopted, a slight exacerbation may be perceived towards the next evening; but this will generally be of brief duration, and will be followed by a complete intermission, when the quinine may be given in doses of ten grains every morning for three or four days.

Although I have described the effect of large doses of quinine in this disease, it will be perceived that I have not proposed to give any name to the group of sequences induced by its action, for the reason that no single term yet designated will denote its peculiar properties; but the term "sedative," approximates more nearly to its proper designation than any other, and has generally been adopted by the advocates of its employment.

I could fill the pages of your journal with extracts from the writings of distinguished physicians of our own country to corroborate what I have stated, but, instead of doing so, must refer the skeptic to our Southern and Western journals.

To those who have observed the action of this remedy in the different modes and various conditions of the system in which it is administered, the inference is irresistible, but its effects are solely dependent upon its salutary action upon the nervous system. This effect is so often immediate, arresting the disease at once, before it could be *absorbed in the blood, or resolve inflammation,* that we are compelled to regard its action as purely nervous. Now if it be contended that this fever is merely symptomatic of gastro-enteric lesion, none will surely argue with Broussais that quinine arrests the febrile movement by its local application to the inflamed surface; that it has any power to effect this, has never been demonstrated, and is opposed to the experience of all. Or if it be contended with Liebig, that in fever some of the elements of the brain are removed which are supplied by the alkaloids of bark, &c., we may reply, that prior to this restoration of the lost elements of the cerebral substance, the agents have to be subjected to the functions of absorption and nutrition, both so imperfectly conducted amid febrile excitement, and for the performance of which a far greater period of time is necessary, even in health, than is amply sufficient for the sanatory action of quinine. These conflicting, but ingenious theories of two great minds, only serve to make the "darkness visible" that surrounds the essential nature of disease and its remedies.

The effect of quinine upon the pulse is one of the most remarkable of its powers in this disease. I have reduced the pulse from 140 and 150 pulsations in a minute to its normal standard in six hours. Indeed, I have in several cases desisted from its continuance, for fear that its sedative effect might be carried too far. The organic lesions, therefore, so often developed in the course of this fever, so far from contra-indicating its use, are certainly relieved by it, or their cure accelerated. Where blood-letting, both general and local, assisted by the other usual antiphlogistic measures, have failed in arresting cerebral inflammation, quinine in a few hours completely arrested the progress of the disease, reducing the pulse, calming delirium, promoting free diaphoresis, and inducing sleep, from which the patient would awake to speedy convalescence, quinine would seem to effect this by allaying the morbid excitement of the nervous system, upon which this increased action of the heart and arteries is dependent, and from the continued excitement of which the organic lesions developed would seem to arise.

It will need but a short experience with this article to convince the careful observer that it possesses the power of allaying nervous irritation, spasm and pain. Neuralgia, whether intermittent or continued, will almost invariably yield to its sedative and anodyne properties.

Those to whom the facts and views herein set forth are entirely novel, may here enquire, "If there is no *danger* in administering quinine during febrile and inflammatory excitement?" Confining my reply to this disease, I unhesitatingly answer, "That there is no danger when given in full and free doses, in the manner which I have indicated!" They may also enquire, "Have all authors on therapeutics committed an error in classing quinine amongst the excitants, in fact, as the first and most valuable of tonics?" To which I reply, that all the standard authorities on this subject I believe to be perfectly correct; but their knowledge of quinine is confined to its tonic, stimulant and antiperiodic character, as it assuredly is in small, repeated doses, but given in large doses it produces the effects I have described; and therefore not only admissible, but indispensable where small doses would not only be improper but pernicious. I speak from great experience with this article. I have given 60 ounces in one fall, (1846,) and I here declare my belief that armed with this agent, remitting fever, if attacked early, is as certainly and completely under its control as ague. I have given it in the chill, the height of the paroxysm and its decline, complicated with indisputable evidences of lesion of the cerebral and abdominal organs without any injurious effect in any case; and to sum up in few words, the sulphate of quinine in full doses allays pain, irregular nervous action and restlessness, diminishes the pulse in frequency, fullness and force, removes local determination and equalizes the circulation, by allaying that morbid excitement of the nervous system upon which remitting fever depends, and consequently relieves the local lesions arising from this exalted action of the circulation.

"Hæc certamina tanta
Pulveris exigini factu compresa quiescunt."

